# A Large-Scale and Serverless Computational Approach for Improving Quality of NGS Data Supporting Big Multi-Omics Data Analyses

**DOI:** 10.3389/fgene.2021.699280

**Published:** 2021-07-13

**Authors:** Dariusz Mrozek, Krzysztof Stępień, Piotr Grzesik, Bożena Małysiak-Mrozek

**Affiliations:** ^1^Department of Applied Informatics, Silesian University of Technology, Gliwice, Poland; ^2^Department of Graphics, Computer Vision and Digital Systems, Silesian University of Technology, Gliwice, Poland

**Keywords:** next-generation sequencing, data quality, cloud computing, big data, data lake, OMICS data, serverless, querying

## Abstract

Various types of analyses performed over multi-omics data are driven today by next-generation sequencing (NGS) techniques that produce large volumes of DNA/RNA sequences. Although many tools allow for parallel processing of NGS data in a Big Data distributed environment, they do not facilitate the improvement of the quality of NGS data for a large scale in a simple declarative manner. Meanwhile, large sequencing projects and routine DNA/RNA sequencing associated with molecular profiling of diseases for personalized treatment require both good quality data and appropriate infrastructure for efficient storing and processing of the data. To solve the problems, we adapt the concept of Data Lake for storing and processing big NGS data. We also propose a dedicated library that allows cleaning the DNA/RNA sequences obtained with single-read and paired-end sequencing techniques. To accommodate the growth of NGS data, our solution is largely scalable on the Cloud and may rapidly and flexibly adjust to the amount of data that should be processed. Moreover, to simplify the utilization of the data cleaning methods and implementation of other phases of data analysis workflows, our library extends the declarative U-SQL query language providing a set of capabilities for data extraction, processing, and storing. The results of our experiments prove that the whole solution supports requirements for ample storage and highly parallel, scalable processing that accompanies NGS-based multi-omics data analyses.

## 1. Introduction

Several commercially available sequencing platforms on the market today allow thousands or even millions of DNA/mRNA sequence fragments (sequence reads) to be obtained simultaneously. Raw data obtained once the sequencing is complete include a set of many short genome sequence reads that usually undergo several phases of data analysis. The NGS data pre-processing scheme preceding a secondary data analysis should include sequence quality control and data processing phase, covering the removal of low-quality sequences and bases, demultiplexing, removal of adapters, primers, and contamination, error correction, and detection of enrichment biases. Each nucleotide in the DNA/mRNA read is accompanied by information about the probability of its misidentification. This probability directly determines the *phred* quality score, which is given for the DNA sequence reads in FASTQ files. The quality score *Q* for a base-call is a logarithmic measure depending on the probability *P* of incorrect nucleotide identification (Ewing and Green, 1998):

(1) $$\begin{array}{l} Q=-10\times \log_{10}P. \end{array}$$

High values of the quality score correspond to low probabilities of misidentification errors, and conversely. Low-quality bases are often located at the very beginning of the sequence. The probability of misidentifying nucleotides also increases with the position in the read. In addition, raw data may be contaminated with fragments of the adapter sequences that do not belong to the sequenced material. Therefore, the quality improvement of NGS sequence reads is vital for further analysis of genomic data analyses since the presence of poor quality or technical sequences may degrade the results of the analyses.

At the same time, as a high-throughput technology, NGS sequencing generates vast amounts of biomedical data. This raises challenges of Big Data (Mrozek, 2014, 2018) not only due to the volume of data generated, but also due to the *velocity* (i.e., speed) in which the data is produced in various projects, and the *variety* of formats in which the data is delivered. These three V characteristics (i.e., volume, velocity, variety), typical for Big Data problems, largely influence the *value* that can be retrieved from the data. The implementation of even small projects that require data from the NGS sequencing of multiple genomes can pose many problems related to the infrastructure necessary to perform the task. The infrastructure must provide the needed storage space and computing power to process large amounts of information efficiently. Therefore, highly distributed and scalable environments are recently used to solve the challenges of NGS Big Data processing and NGS-based analyses performed at various steps of analysis workflows, from primary to tertiary.

These environments rely on a broad Hadoop ecosystem and its tools. For example, SeqPig (Schumacher et al., 2013) as a dedicated library for distributed analysis and processing of large NGS sequencing data on Hadoop clusters extends the processing capabilities of Apache Pig and the Pig Latin scripting language. Apart from processing files in FASTA and FASTQ formats, the library enables the assessment of the quality of sequences. Several Hadoop-based solutions were proposed for the secondary NGS data analysis steps, including initial alignment of short reads to a reference genome with BigBWA tool (implementing the Burrows-Wheeler Aligner (Abuín et al., 2015), tag SNPs selection (Hung et al., 2015), and construction of phylogenetic trees based on ultra-large DNA sequences (Zou, 2016; Zou et al., 2016). Within the tertiary analysis of NGS data, the GenoMetric Query Language (GMQL) (Masseroli et al., 2015, 2018) simplifies the variant analysis in genomic data stored in Hadoop Distributed File System (HDFS) with a declarative query language, distributed processing, and integration of heterogeneous biomedical data sources (Masseroli et al., 2016). Furthermore, Wiewiórka et al. (2019) proposed a library for scalable depth of coverage calculations over genomic data on Apache Spark. These solutions prove that distributed processing can solve the problems of voluminous and quickly produced data.

On the other hand, the *variety* of data, which next to the *volume* is one of the challenges affecting the NGS data obtained in several formats after particular phases of data production, processing, and analysis, causes the need for efficient and scalable data storage. Big Data lakes that allow storing the data before and after data analyses in the native formats facilitate gathering all the data in one place. However, processing the data must be accompanied by specific steps of data extraction. We first introduced the Extract, Process, and Store (EPS) process in Małysiak-Mrozek et al. (2018) for processing biomedical data with the use of fuzzy techniques. It clearly exposed the extraction and storing phases that can also be parallelized while processing big data in a distributed manner.

We adopt this idea in the NGS data processing performed in this paper to improve processing performance for large amounts of NGS data while at the same time reducing the operational overhead by taking advantage of the serverless nature of the Data Lake Analytics service. However, we also show limitations of the used data lake platform and the EPS while processing NGS data.

### 1.1. Related Works

The growing body of research shows that the quality of NGS data is important for future NGS-based multi-omics data analyses. There are many approaches and tools dedicated to processing and cleaning the DNA/RNA sequences obtained with single and paired-end sequencing techniques in the literature. First of them, Trimmomatic, introduced by Bolger et al. (2014), is a tool dedicated to trimming and filtering next-generation sequencing reads, supporting both single and paired-end reads. For trimming, it offers two algorithms, one called “simple,” which tries to find an approximate match between provided adapter sequence and read, and the second, called “palindrome mode,” which is dedicated to detecting contaminants at the end of the reads. It also offers to filter sequences based on Illumina quality score. According to performance experiments presented in the paper, it is faster than comparable tools such as AdapterRemoval, Reaper, or Cutadapt. Schubert et al. (2016) propose AdapterRemoval v2, an improvement to an AdapterRemoval introduced previously in Lindgreen (2012). It is a tool that allows for the trimming of adapter sequences from both single-end and paired-end FASTQ reads. It takes advantage of a modified Needleman-Wunsch algorithm (Needleman and Wunsch, 1970). Additionally, it also allows for the merging of overlapping paired-ended reads into consensus sequences. According to the performance experiments presented in the paper, it offers performance comparable to Trimmomatic. Another tool that takes advantage of the Needleman-Wunsch algorithm has been introduced by Roehr et al. (2017). The authors present FLEXBAR 3.0, an improvement to previously introduced FLEXBAR (Dodt et al., 2012), which is a sequence trimming software dedicated to processing NGS reads and trimming barcode and adapter sequences. It supports five trimming modes, LEFT, LEFT-TAIL, RIGHT, RIGHT-TAIL, and ANY. In version 3.0, it introduced multi-threading and SIMD vectorization to improve performance over previous versions. According to benchmarks presented by the authors, it offers better trimming quality than Trimmomatic but takes two times longer to process the same number of reads. Criscuolo and Brisse (2013) introduced AlienTrimmer, a tool dedicated to the removal of alien sequences such as primer, adapters, or barcode sequences from raw next-generation sequencing data. The tool supports the removal of such sequences from both 5′ and 3′ ends. It uses an algorithm based on the k-mer decomposition of specified alien sequences and then tries to find occurrences of such k-mers in the sequence. The authors highlight that k-mer decomposition-based algorithms, such as the one used in AlienTrimmer, are prone to decreased accuracy in case of sequencing errors and when handling short fragments of alien sequences. Another tool dedicated to trimming adapters and low-quality bases in next-generation sequencing data, Btrim, has been introduced by Kong in Kong (2011). When performing adapter trimming, the tool uses an algorithm that is based on a modified version of Myers' bit-vector dynamic programming algorithm. When performing trimming of bases with low quality, it switches to a moving window algorithm that trims bases if the average quality score is lower than the predefined threshold. It supports data in FASTQ for both Sanger and Illumina reads. Smeds and Künstner (2011) proposed ConDeTri, a content-dependent trimming solution dedicated to processing and trimming Illumina reads. It supports the removal of sequencing errors from the 3' end as well as the removal of reads with low-quality bases. The algorithm allows keeping low-quality bases (below the threshold) if they are surrounded by high-quality bases. Martin, in his work (Martin, 2011), introduces Cutadapt, which is another tool dedicated to removing adapter sequences from Illumina reads. Cutadapt trims at most one adapter sequence in a single run and does not offer other trimming capabilities. According to the benchmarks presented in Schubert et al. (2016), it offers slower performance than AdapterRemoval and Trimmomatic. Unlike Cutadapt, PEAT (Paired-End Adapter Trimmer) (Li et al., 2015) does not require providing adapter sequence but instead detects adapter sequence by finding mutually reverse-complement region between paired reads. It is also not capable of processing barcode sequences on 5' ends, does not take the read quality scores into account, but for benchmarked datasets, it offered much better performance in terms of speed than tools such as AdapterRemoval and Trimmomatic. For trimming paired-end NGS reads, Skewer (Jiang et al., 2014) adapter trimmer offers better memory efficiency while being slower than solutions like Trimmomatic and Btrim.

In addition to tools that are dedicated mostly to trimming adapter sequences, there are also toolkits, like Kraken (Davis et al., 2013), FASTX-Toolkit (Gordon, 2008), or ERNE (Del Fabbro et al., 2013), that allow building advanced pipelines for analyzing NGS data, where filtering and adapter trimming is only one of the steps. In terms of the declarative nature of the adapter trimming, Fuzzysplit, a flexible fuzzy search library (Liu, 2019) provides a pattern language that can be used to define adapter patterns that should be detected in target sequences. However, it does not support any other matching algorithms and does not consider quality scores from FASTQ formats. It offers great flexibility at the cost of a steep learning curve and the requirement to write custom templates for each supported format.

In terms of addressing Big Data challenges, Expósito et al. (2020) proposed SeQual for large-scale processing of NGS reads on Apache Spark. It implements filtering, trimming, and formatting procedures, operates on FASTQ and FASTA data formats, and offers a user-friendly graphical user interface. However, it requires access to the Spark computational cluster.

While there are also other local tools dedicated to trimming NGS data, such as ea-utils (Aronesty, 2011, 2013), PRINSEQ (Schmieder and Edwards, 2011), SeqPurge (Sturm et al., 2016), PE-Trimmer (Liao et al., 2020), StreamingTrim (Bacci et al., 2014), AfterQC (Chen et al., 2017), ClinQC (Pandey et al., 2016), UrQT (Modolo and Lerat, 2015), pTrimmer (Zhang et al., 2019), Fastq_clean (Zhang et al., 2014), they are often designed as separate programs instead of libraries and only one of them, Fuzzysplit, offers declarative interface, but has limited functionality. They are often also not designed for Big Data processing that takes advantage of Cloud Computing technologies, except SeQual, which is built on top of the Apache Spark framework. The downside of SeQual is that the underlying Apache Spark cluster has to be provided and managed, which adds operational complexity and requires knowledge about managing the computing cluster itself.

### 1.2. Scope of the Work

It is worth noting that most of the works mentioned in previous sections do not focus on improving the quality of NGS data at a large scale. Moreover, only one of them provides declarative querying capabilities for this purpose but with limited NGS data quality improvement capabilities. Our solution hybridizes different technological approaches, which finally leads to possessing three fundamental properties—it is declarative, addresses challenges of Big NGS Data, and is scalable on the Cloud. Moreover, unlike SeQual, it does not require complex management of the computational cluster.

To solve the problems of Big NGS Data, in this paper, we present the scalable solution that utilizes the Data Lake ecosystem and serverless computing on the Microsoft Azure platform, enabling NGS data cleaning in the Cloud. Furthermore, we show how we can use the Data Lake ecosystem to build an environment for distributed storing and analyzing NGS data. This will be demonstrated by implementing solutions designed to control and improve the quality of reads from raw data. The results of our experiments show that the storage method and the degree of parallelism have the most significant impact on the time necessary to pre-process the sequence in terms of their quality improvement and thus on the costs of using the Cloud platform incurred.

## 2. Materials and Methods

The approach we propose for big NGS data cleaning assumes storing the genomic data in NGS data lake in the Azure Data Lake Store in Microsoft Azure cloud and performing serverless but highly scalable processing of the data by formulating processing queries in the declarative U-SQL language. The data lake is the place where data can be stored in its original format, including structured, semi-structured, and unstructured data. This allows applying the *schema on read* approach while processing the same data for various purposes. In contrast to the *schema on write* approach widely used in transactional systems, the *schema on read* approach schematizes the data when it is needed. Furthermore, the U-SQL language combines the declarative nature of the SQL language with imperative capabilities of C# programming language to process data in a scalable manner, which fits the scenario of big NGS data processing. Finally, serverless computing allows skipping the management of the servers responsible for data processing and frees the user from keeping the running servers all the time (which usually increases costs). By applying the serverless approach, we rely on the computing resources that are allocated by the cloud provider only when we need to execute the processing jobs.

**Figure 1:**
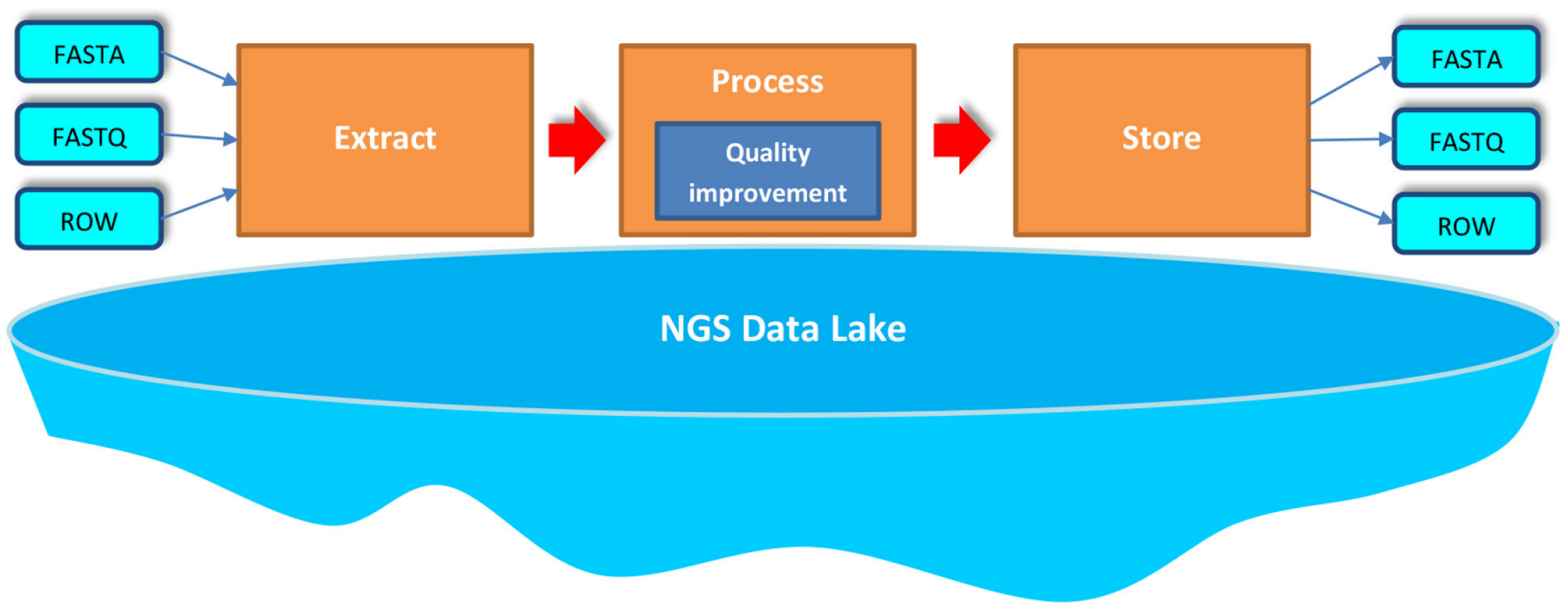
Extraction, processing, and storing (EPS) over big NGS data lake.

In our approach, we process big NGS data stored in NGS Data Lake in three phases—Extract, Process, and Store (EPS)—as it is shown in Figure 1. Particular phases of the EPS allow for the following:

1. Extract—uses various *extractors* to extract appropriate data stored in the data lake, read it, and load the data for further processing,
2. Process—applies developed *processors* for NGS data to perform a set of transformations on the extracted NGS data set; these transformations cover the process of improving the quality of data,
3. Store—uses various *outputters* to store the processed data back in the NGS data lake.

### 2.1. NGS Data Extraction

Data extraction allows reading data from the specified files in the data lake. General workflow for data extraction from a single NGS data file is presented in Algorithm 1. Standard files (e.g., in FASTQ format) are extracted as a whole (by a single computational unit, called Allocation Unit or AU)—lines 8–12. Large files in the row-oriented format (see later in this section) are additionally split into smaller chunks and extracted in parallel. In both cases, for each DNA sequence read *r*_*j*_ in the file or chunk, the extractor *E* extracts the data appropriately (depending on the format) and represents it in the row-oriented format. The sequence read in a row-oriented format $r_{j}^{T}$ is added to the data chunk c^*^ (a resultant rowset, line 5 and 10).

The symbol *T* (line 10) denotes transposition, and we use it when the NGS data is extracted from FASTQ files, where each DNA sequence read *r*_*j*_ is represented by a quadruple:

(2) $$\begin{array}{l} r_{j}=\left[\begin{array}{c} d_{1}\\ s\\ d_{2}\\ q \end{array}\right], \end{array}$$

where *d*_1_ contains sequence identifier and an optional description, *s* is a raw sequence, *d*_2_ is a separator line beginning with a plus (+) sign with an optional description, *q* contains encoded quality scores for base calls in the sequence *s*.

**Algorithm 1:**
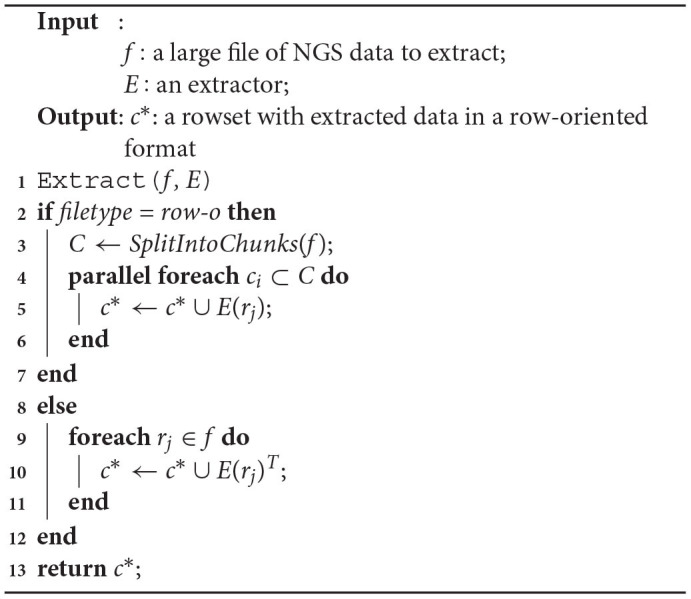
NGS data extraction for a single file located in big NGS data lake.

Files in the FASTQ format contain many of such reads and can be represented as:

(3) $$\begin{array}{l} f_{FASTQ}=\left[\begin{array}{c} r_{1}\\ r_{2}\\ \ldots \\ r_{\left|f\right|} \end{array}\right], \end{array}$$

where |*f*| denotes the number of sequence reads in a file. The extraction process is a function that temporary changes the format of the data to the row-oriented one:

(4) $$\begin{array}{l} \left[\begin{array}{c} r_{1}\\ r_{2}\\ \ldots \\ r_{\left|f\right|} \end{array}\right]\overset{E}{\to }\left[\begin{array}{c} r_{1}^{T}\\ r_{2}^{T}\\ \ldots \\ r_{\left|f\right|}^{T} \end{array}\right]. \end{array}$$

For the paired-end sequencing, we operate on two files with forward (left) and reverse (right) sequence reads

(5) $$\begin{array}{l} f_{FASTQ}^{f}=\left[\begin{array}{c} r_{1}^{f}\\ r_{2}^{f}\\ \ldots \\ r_{\left|f\right|}^{f} \end{array}\right]\text{ and }f_{FASTQ}^{r}=\left[\begin{array}{c} r_{1}^{r}\\ r_{2}^{r}\\ \ldots \\ r_{\left|f\right|}^{r} \end{array}\right], \end{array}$$

where $r_{j}^{f}$ and $r_{j}^{r}$ are corresponding forward (left) and reverse (right) sequence reads. Therefore, the extraction process provides an appropriate row-oriented representation for them that looks as follows:

(6) $$\begin{array}{l} f_{ROW-O}^{paired}=\left[\begin{array}{cc} r_{1}^{f} & r_{1}^{r}\\ r_{2}^{f} & r_{2}^{r}\\ \ldots & \ldots \\ r_{\left|f\right|}^{f} & r_{\left|f\right|}^{r} \end{array}\right]. \end{array}$$

If there are many files with independent genomic data to be extracted or in case a large genomic data file is divided into smaller files (e.g., intentionally), the extraction process can be further parallelized on many Allocation Units (Algorithm 2, line 1). Each file *f*_*l*_ in a collection of files *F* undergoes the same extraction steps (line 2) as in Algorithm 1. This produces many rowsets *c*_*l*_ in the row-oriented format that are either independent partitions of data for multiple genomic data files or are merged in a single rowset, when operating on many smaller files for one sequencing experiment (line 3). The collection of rowsets or a merged rowset *C* is then returned for further processing in the Process phase of the EPS (line 5).

**Algorithm 2:**
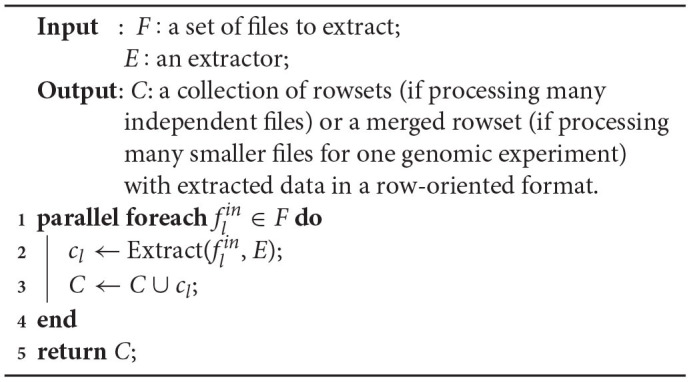
Parallelization of NGS data extraction from files located in big NGS data lake.

Reading data from files located in the NGS data lake is implemented in the EXTRACT expression of the U-SQL language. The EXTRACT expression consists of a list of attributes extracted, a FROM clause followed by a file path, and a USING clause followed by an instance of the extractor that defines how the files should be read (like in Listing 1). The library that we developed allows extraction from three file formats used to store raw NGS data. With the library, we can read data from FASTQ file format, dedicated to storing NGS raw data. Additionally, we designed a dedicated row-oriented format for processing NGS data on the Azure Data Lake platform, which improves the performance of the processing. The new data format assumes that all data related to one sequence is kept in a single row, in sections separated by a delimiter, which is a vertical bar “|.” This format was specially designed during the implementation of this work to make the best use of the possibilities of the Data Lake services. The layout of a single row that stores information describing the corresponding reads (paired-end) in the row-oriented file is shown below and implements the representation from formula 6.

<Description of read 1>|<Sequence 1>|<Optional descr.>|

<Quality values for read 1>|

<Description of read 2>|<Sequence 2>|<Optional descr.>|

<Quality values for read 2>|

Consequently, for the new row-oriented format, we also implemented appropriate extractors that enable reading NGS data stored in it. We also provided the ability to read data from FASTA format files. However, files in this format do not store information on the sequence reads quality. Therefore, no mechanism for cleaning data stored in this format has been implemented in the Process phase. Simple operations on FASTA files can be performed using U-SQL expressions (shortening the sequence to a specific length, removing short sequences, etc.). In summary, the following extractors were prepared for reading NGS data:

- FastaExtractor—for reading data from files in the FASTA format.
- FastqExtractor—for reading data from files in the FASTQ format. As an argument, the extractor takes a *Boolean* value that indicates whether the identifier taken from the first description line of a read should be written to a separate column. By default this value is set to *true*.
- FormattedFastaExtractor—for reading from files in a row-oriented version of the FASTA format.
- FormattedFastqExtractor—for reading from files in a row-oriented version of the FASTQ format.
- FormattedPairedEndExtractor—for reading data from files in the row-oriented version of the FASTQ format, in which data from paired-end sequencing related to a single read are stored in one row of the file.

**Figure 2:**
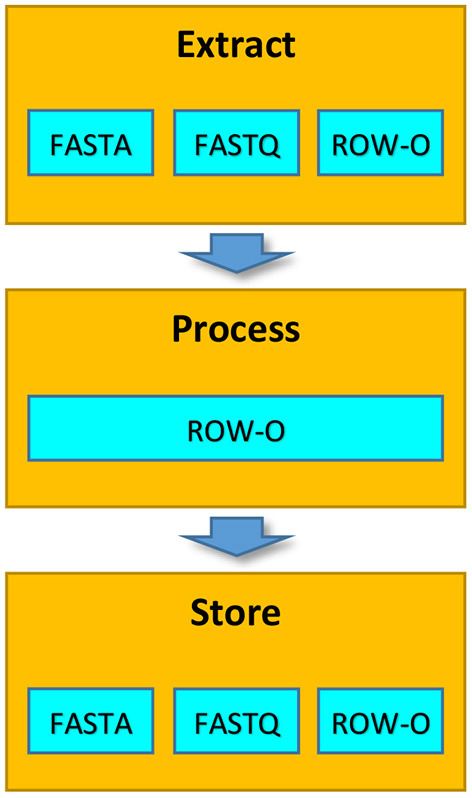
Formats of data representation in particular phases of the EPS process.

It is worth noting that although before extraction the data can be stored in various formats, in the Process phase, the extracted data is always represented in the row-oriented format (Figure 2). This representation allows processing the data more efficiently (see section 2.4 for details).

Examples of data reading with the use of the implemented extractors are presented in Listing 1. U-SQL enables reading data in parallel from multiple files located in a given location. Information on sequence reads resulting from paired-end sequencing is usually stored in two separate FASTQ files (like in Listing 1, lines 3 and 8). In order to be able to process such data in the successive steps, it is required to link the corresponding reads from these two files (lines 11–16).

**Listing 1:**
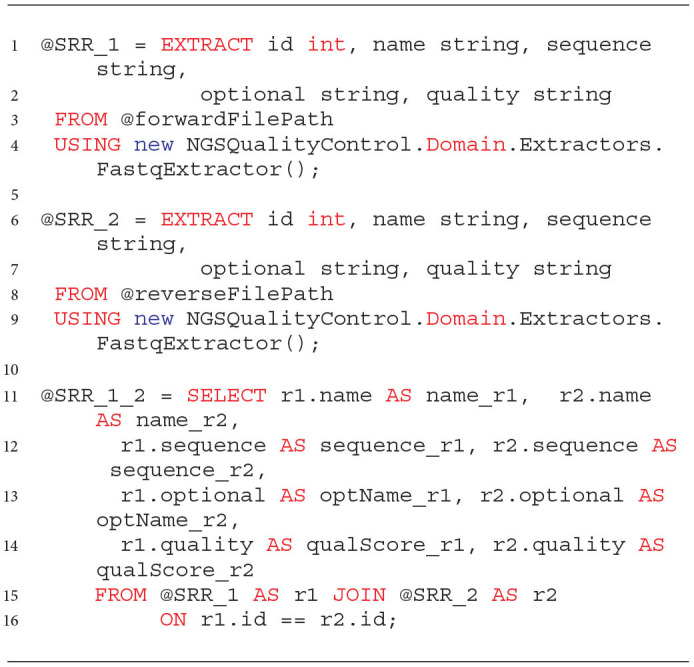
Reading data from two files and linking reads related to the same sequence.

The Extraction process can be quite complex, and the invocation of extractors according to the U-SQL syntax may cause troubles for those users and NGS analysts who are not familiar with programming. Therefore, to facilitate using the above-mentioned solutions, we added wrapping functions that enable the same functionality of reading NGS data. Examples of these functions are presented in Listing 2.

**Listing 2:**
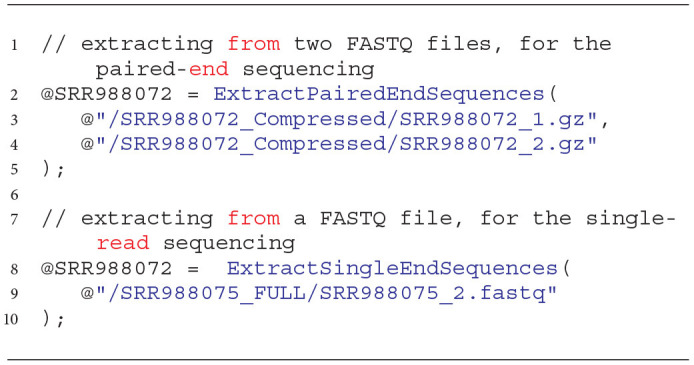
Invocation of wrapping functions for extraction of data from FASTQ files with data obtained with the paired-end and single-read sequencing techniques.

### 2.2. NGS Data Processing: Improving NGS Data Quality

NGS data processing covers applying a set of transformations for the rowset generated in the Extract phase. The phase is parallelized for large rowsets *c* provided at the input (Algorithm 3). First, the rowset *c* is divided into many data chunks (line 2). Then, each data chunk *c*_*i*_ is processed in parallel on allocation units by applying cleaning transformations *t*_*k*_ ∈ *T*_*R*_ for each row (sequence read in a row-oriented format) *r*_*j*_ of the data chunk *c*_*i*_. The cleaning covers single reads in the single-read mode (lines 5–11) or forward (left) and reverse (right) reads in the paired-end sequencing mode (lines 12–18). Results are stored in the new rowset $c_{i}^{*}$ (lines 10 and 17). At the end, all new data chunks are merged together into new rowset c^*^ with cleaned data (line 21, |*C*| is the number of data chunks the input rowset *c* was divided into).

**Algorithm 3:**
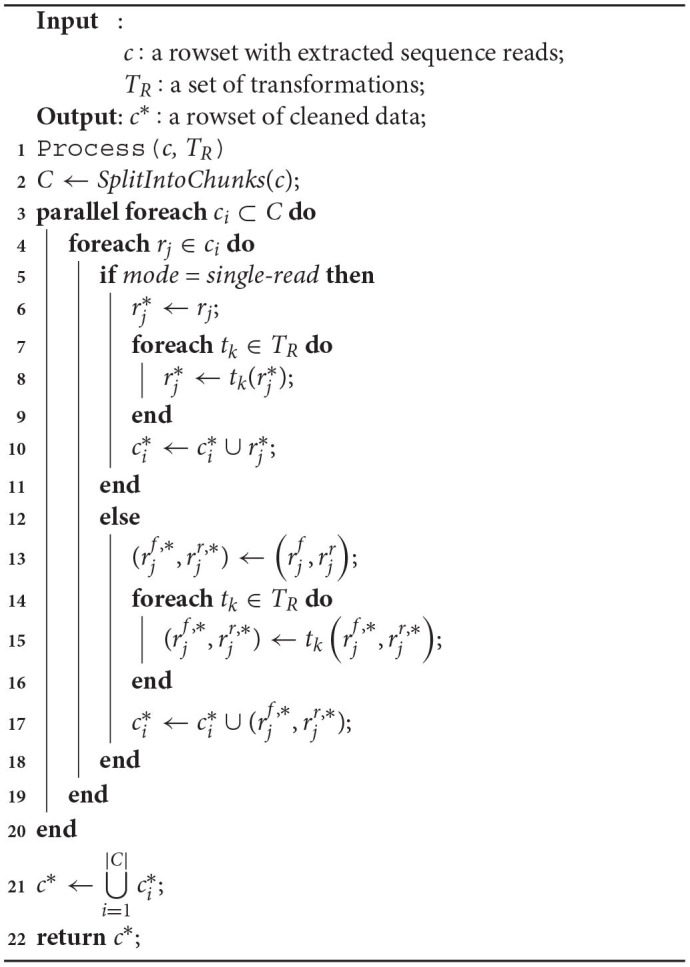
Processing a row-oriented data partition (a rowset) from a single NGS data file (or a pair of files for the paired-end sequencing).

Improving NGS data quality is implemented in the U-SQL and performed through a set of transformations implemented in the Process phase of the EPS process. The set of transformations is modeled based on the capabilities of the Trimmomatic tool (Bolger et al., 2014). Trimmomatic works in two modes: single-read and paired-end. We have implemented the following commands for improving data quality in our tool:

- ILLUMINACLIP—removes Illumina adapters from sequence reads,
- SLIDINGWINDOW—removes nucleotides using the sliding window method; starts scanning at the 5' end and cuts off the read when the average quality in the window falls below the threshold value,
- MAXINFO—removes nucleotides with an adaptive method, by balancing the read length and error level to maximize the quality of each read,
- LEADING—removes nucleotides from the beginning of the sequence as long as their quality is lower than the specified threshold,
- TRAILING—removes nucleotides from the end of the sequence as long as their quality is below the specified threshold,
- CROP—reduces reads to a specified length,
- HEADCROP—deletes the specified number of nucleotides from the beginning of the read,
- TAILCROP—deletes the specified number of nucleotides from the end of the sequence,
- MINLEN—deletes the read if its length is shorter than the specified value,
- AVGQUAL—deletes the sequence if the average quality of its nucleotides is lower than the specified threshold.

NGS data transformations are performed by invoking dedicated *processors* for the U-SQL queries that are used for parallel processing in the Data Lake environment. We developed two data processors that allow cleaning the NGS reads:

1. FastqPairedEndTrimmerProcessor (wrapped by the ProcessPairedEnd processing function)—allows processing sequence reads obtained as a result of the paired-end sequencing.
2. FastqSingleEndTrimmerProcessor (wrapped by the ProcessSingleEnd processing function)—allows processing sequence reads obtained as a result of the single-read sequencing.

An example of how to process the extracted rowset with developed processors is shown in Listing 3. The processing statement consumes the processed data set with extracted sequence reads and quality information in the PROCESS clause (lines 2 and 10) and generates a new rowset with cleaned NGS sequence reads (lines 1 and 9). The rowset consists of information specified in the PRODUCE clause (lines 3 and 11). Processing is performed with the use of one of the two data processors invoked in the USING clause. These processors accept several arguments. The first one is the String value with a list of cleaning commands (@command, lines 6 and 16). Commands are issued in the order in which they are given. Command arguments are given after the colon symbol “:.” It is recommended that the removal of adapters from NGS reads be performed first. The @illuminaAdaptors argument (defaults to *null*) is a String value that takes the location to the file with adapters to be removed from the input sequences. The last argument takes the quality score coding (PHRED33—set by default, or PHRED64). The @keepUnpaired argument of the Boolean type (for the paired-end data processor) is used to set the flag (*false* by default), forcing the storage of reads that, as a result of cleaning, were deprived of the associated read stored in the second (paired) file.

**Listing 3:**
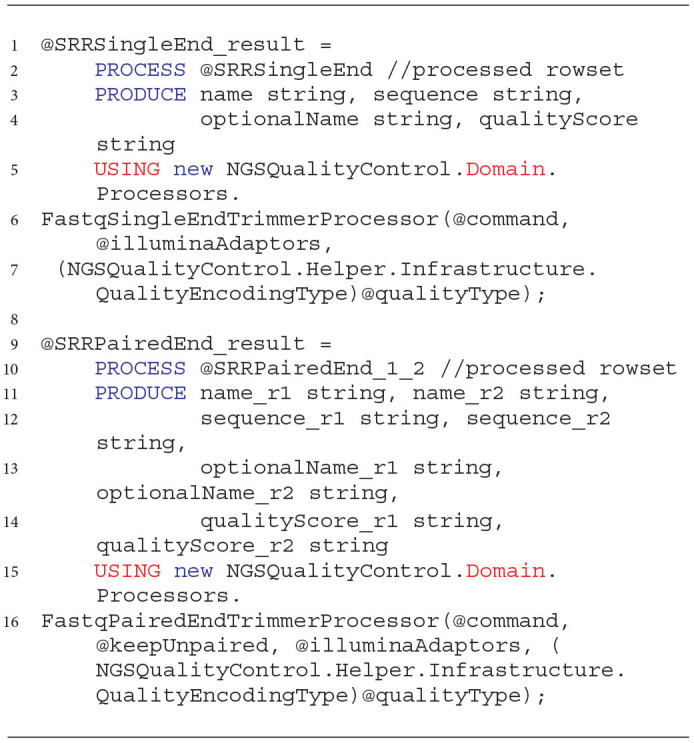
Cleaning NGS data with the developed processors in U-SQL.

As in the case of the extractors used for reading the NGS data, also for the processing phase, we developed the wrapping functions that simplify the use of prepared solutions. These functions and an example of how to use them are presented in Listing 4. They accept the rowset with extracted NGS data as a first argument (lines 2 and 10) and a set of cleaning transformations (commands) as a second argument (lines 3 and 11). The third argument for the paired-end sequencing data processor tells it what to do with the reads left unpaired after the cleaning (the DEFAULT value means not to keep them, line 4).

**Listing 4:**
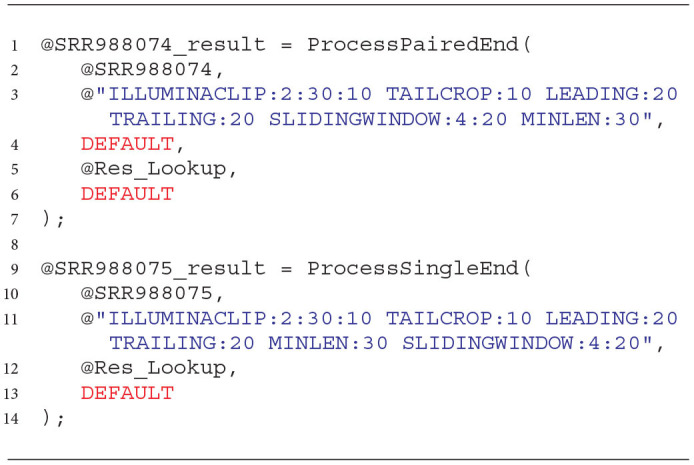
Wrapping functions for cleaning NGS data with the developed processors in the Data Lake environment.

The last two arguments correspond to the location of the dictionary of adapters to be removed (@Res_Lookup, lines 5 and 12) and the quality score encoding (lines 6 and 13, DEFAULT means PHRED33). Both functions return cleaned rowsets of NGS data.

### 2.3. NGS Data Storing

Storing data completes the EPS process for the NGS data. It is performed according to Algorithm 4. The procedure accepts the rowset *c* with the processed data, a dedicated outputter *O*, and the name of the output file (or files, depending on the mode). The rowset is split into several data chunks (line 2) that are written into several parts of the file(s). The offset is determined by the data chunk *c*_*i*_ (lines 6 and 9–10). The degree of parallelism depends on the size of data written, the used outputter, and the maximum number of AUs specified by the user while executing the job. Custom outputters (storage processors) may, however, serialize this part of the EPS (see Table 1). Each read *r*_*j*_ in the rowset is stored appropriately depending on the destination format specified (e.g., it is transposed again to be represented in the FASTQ format, unless we use the row-oriented format to store the data in the output files).

**Algorithm 4:**
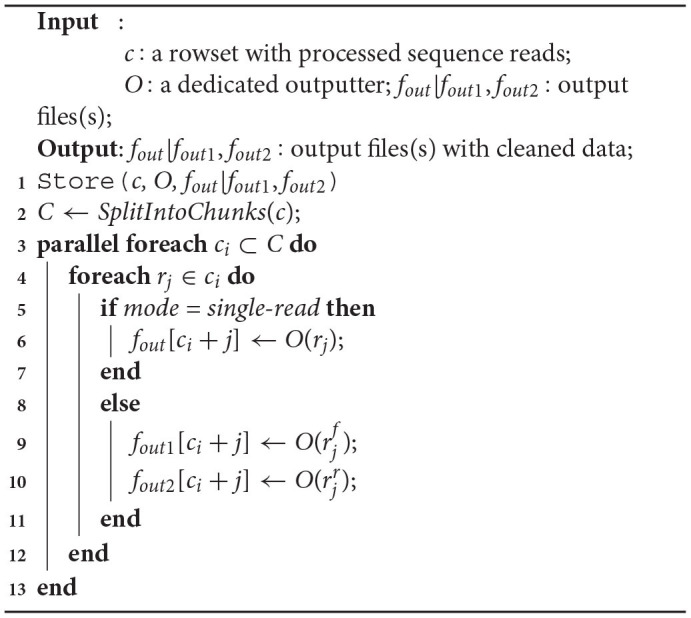
Storing a processed rowset of NGS data to output files with a dedicated outputter *O*.

**Table 1:** Granularity levels of parallel computations used for particular storage formats in particular phases of the EPS process.

	**FASTA**	**FASTQ**	**Row-O**
Extract	Coarse- or Medium-grained	Coarse- or Medium-grained	Medium-grained
Process	Medium-grained	Medium-grained	Medium-grained
Store	Coarse-grained	Coarse-grained	Medium-grained

The Store phase implemented in U-SQL covers saving the output of processing scripts to a file in the Data Lake or a database. The data is written to the file using one of the dedicated outputters that we have developed for various formats that NGS data can be stored in. Five different output interfaces have been prepared for this purpose:

1. FastaOutputter—saves data to a file in the FASTA format,
2. FastqOutputter (with the SavePairedEndRowsetDecompressed and the SaveSingleEnd-RowsetDecompressed functions)—saves data to a file in the FASTQ format,
3. FastqGzipOutputter (with the SavePairedEndRowsetCompressed and the SaveSingleEnd RowsetCompressed functions)—saves data to a compressed FASTQ file.
4. FormattedFastqOutputter (with the SaveFormattedPairedEndRowsetDecompressed and the SaveFormattedSingleEndRowsetDecompressed functions)—saves data to a file in the row-oriented version of the FASTQ format; as an argument, it uses a Boolean value that specifies whether the rowset being stored contains reads resulting from paired-end sequencing or only reads from single-read sequencing,
5. FormattedGzipFastqOutputter (with the SaveFormattedPairedEndRowsetCompressed and the SaveFormattedSingleEndRowsetCompressed functions)—stores data to the compressed, row-oriented version of the FASTQ format.

An example of invoking the proposed outputters to store NGS data after the quality improvement to a file in the Data Lake is presented in Listing 5. The OUTPUT clause accepts the rowset with the cleaned NGS data that will be stored (lines 1 and 5). The NGS data will again be stored in the Data Lake in the destination file specified in the TO clause either directly or by a string variable (lines 2 ad 6). Finally, the data are persisted in the storage space in a specific format by invoking a particular outputter (lines 3 and 7).

**Listing 5:**
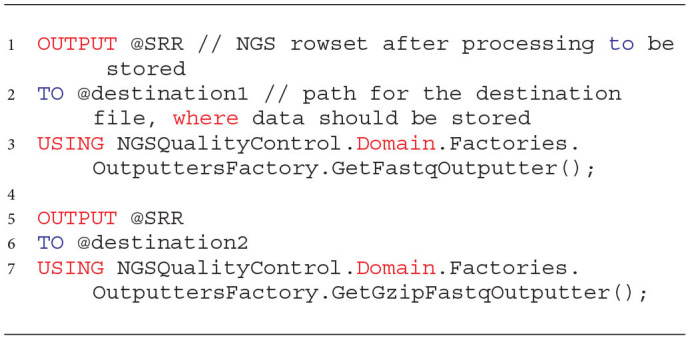
Sample invocations of the Store phase for two developed outputters saving cleaned NGS data in the uncompressed and compressed FASTQ formats.

To unify the coding related to saving the processed NGS data to a file in the Data Lake, for each of the outputters, we also created wrappers that facilitate the use of implemented mechanisms. Sample functions for storing paired-end NGS data as decompressed and compressed files are presented in Listing 6. As arguments, the wrapping functions take paths to the files to which the NGS data from the NGS resultant rowset should be saved (lines 3–4 and 10–11). The rowset with cleaned data is given as the last argument (lines 6 ad 12).

**Listing 6:**
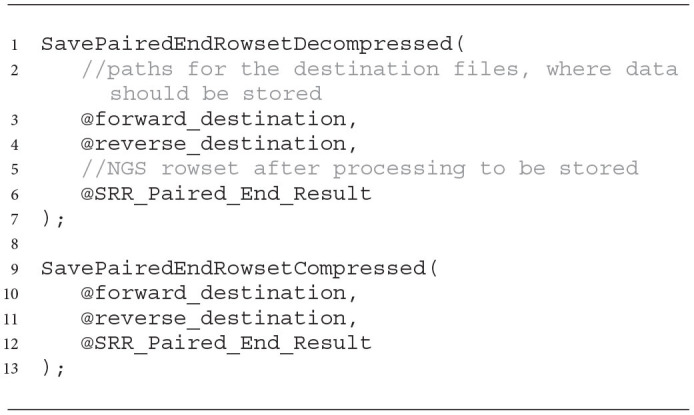
Sample wrapping functions for storing paired-end NGS data after processing and improving its quality.

### 2.4. Granularity of Parallelism

Parallel computations can be performed according to various levels of granularity, including fine-grained, medium-grained, and coarse-grained. The granularity defines the amount of computational work performed within a single task. While performing the quality control and NGS data cleaning in the proposed Data Lake environment, we can apply two types of parallelism:

- Coarse-grained parallelism—this granularity applies when multiple, whole FASTA and FASTQ files are processed in compressed and decompressed form.
- Medium-grain parallelism—this granularity applies when multiple large NGS data from FASTA and FASTQ files are divided into many (*d*) smaller files (e.g., 250 or 750 MB), or when NGS data are stored as a whole in the row-oriented format (then, the splitting is done automatically).

**Figure 3:**
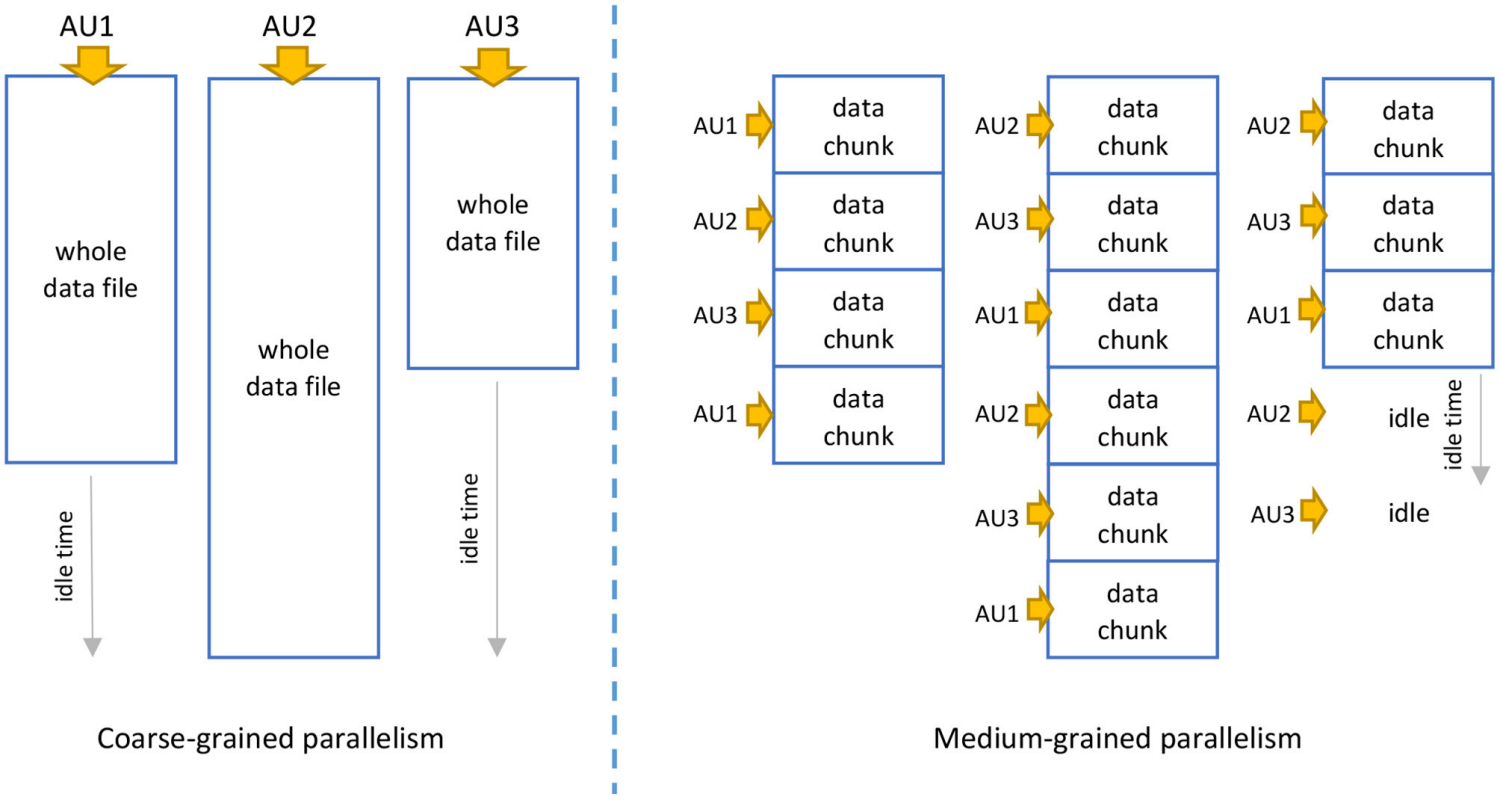
Granularity of parallelism applied in various storage scenarios: **(left)** NGS data processed as a whole by many Allocation Units (AUs), **(right)** processing NGS data divided into chunks.

Both levels of granularity are presented symbolically in Figure 3. In our solution, the granularity of parallelism depends on the format and sizes of processed data files. In the most typical scenario, when whole NGS data files are uploaded to the data lake in the FASTQ format, coarse-grained parallelism will occur. The coarse-grained parallelism relies here on processing individual NGS data files by Allocation Units (AUs) responsible for data processing-related computations. Figure 3 shows only three AUs in action, but there can be many more. This level of granularity is applied due to the large sizes and non-standard format of the NGS data files from the viewpoint of processing data in Big Data environments. Each sequence read entity is composed of four successive rows. This requires dedicated extractors and outputters to extract the data before and store the data after processing. Unlike those used for standard row-oriented data, these are not standard extractors and outputters, where each row constitutes an independent entity. The efficiency of such an approach is lower due to longer idle times resulting from uneven sizes of processed data files.

The average idle time for the coarse-grained parallelism $T_{C}^{idle}$ can be calculated as follows:

(7) $$\begin{array}{l} {\bar{T}}_{C}^{idle}=\frac{1}{n-1}\overset{n-1}{\underset{i=1}{\sum }}\left(T_{max}-T_{AU_{i}}\right), \end{array}$$

where *T*_*max*_ is the longest processing time registered (usually the execution time noted when processing the largest NGS data file), which is equivalent to the execution time of the whole parallel processing, *T*_*A*_*U*__*i*__ is the processing time of the *i*-th NGS data file (another than the largest one) by another AU (other than the one that processes the longest), *n* is the number of AUs in use.

Splitting FASTQ data into multiple data chunks *d* causes changing the granularity of parallelism from coarse-grained to medium-grained and usually increases overall efficiency. This should be visible, especially if the sizes of processed data files differ much. This increase in efficiency is possible due to shorter idle times for AUs that have nothing to process in the final iteration of data processing (assuming that *n* < *d*, we have to perform several processing iterations with the same AUs for different data chunks).

The idle time for the medium-grained parallelism *T*^*idle*^ is equal to the idle time of any AU ($T_{AU_{i}}^{idle}$) processing a data chunk:

(8) $$\begin{array}{l} T^{idle}=T_{AU_{i}}^{idle}. \end{array}$$

The best performance can be achieved when the number of allocated AUs is equal to the number of data chunks (*n* = *d*). Theoretically, in such a case, the idle time *T*^*idle*^ = 0. The number of allocated AUs can be even greater than the number of data chunks (*n* > *d*), but it would unnecessarily increase the cost of using the NGS data lake platform as some AUs would have nothing to process (overallocation).

It is worth noting that medium-grained parallelism is automatically applied when the NGS data is stored in a row-oriented format. This is a non-standard format to keep the NGS data in but, at the same time, a standard format for processing data on Big Data platforms. This fact causes that, unlike in previous cases, the medium-grained parallelism occurs in all phases of the EPS process, including extraction and storing. And this is the reason why we proposed a new format to store the NGS sequence data.

Table 1 summarizes the granularity levels used for particular storage formats in particular phases of the EPS process. When processing row-oriented files, we operate on the medium-grained level of parallelism in all phases of the EPS. For native formats (FASTA and FASTQ), we usually operate on the coarse-grained level of parallelism while extracting and storing the data. This is because we use non-standard extractors. Medium-grained parallelism is achievable in the Extract phase when we pre-process the files and physically divide them into many smaller files. This should speed up the Extract phase but requires an additional preparation step.

## 3. Experimental Results

The presented Data Lake-based approach was tested to verify the quality of results and performance of the NGS data cleaning. We performed tests in the highly parallel Azure Data Lake environment and on local workstations. For the Data Lake environment, we executed the EPS process on the varying number of Allocation Units (AUs).

The purpose of the experiments was to find a way to store NGS data in the Data Lake Store to make the most efficient use of the Data Lake Analytics performing the EPS process, thereby reducing the analysis time and, indirectly, the associated costs of using the scalable platform. In the following sections, we will also briefly present a comparison of the duration of data processing in the cloud and on desktop computers. On the other hand, we also checked the correctness of the obtained results. We checked whether the NGS data processed and cleaned with the use of the developed library are identical to those obtained as a result of analogical processing performed on local workstations with the Trimmomatic program.

During our tests on improving the quality of NGS data, we executed the U-SQL script that looked like the one presented in Listing 7 (executed scripts differed only with the paths to data files extracted and stored as we worked with different data sets). The presented U-SQL script extracts NGS data obtained by means of the paired-end sequencing technique, stored in two files. Then, it processes the files according to the cleaning commands given. Finally, it saves the processed reads to two uncompressed FASTQ files.

**Listing 7:**
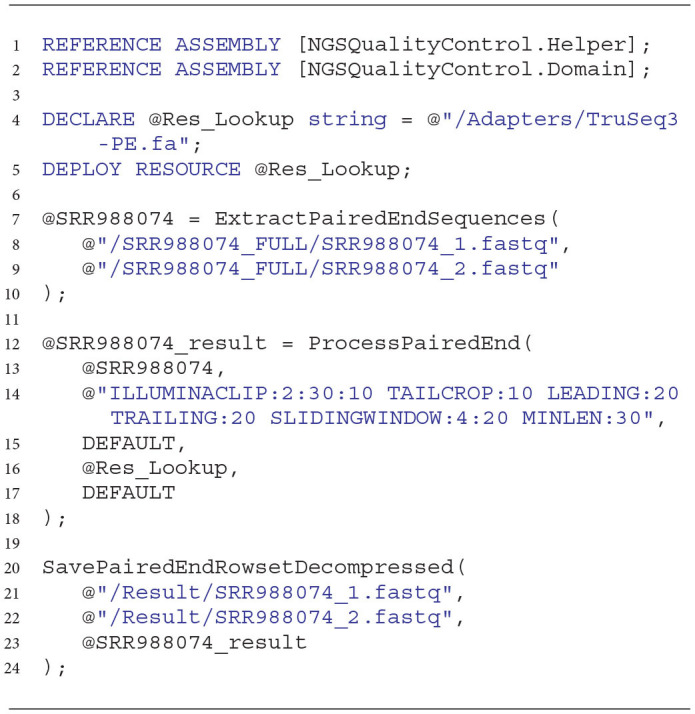
Sample U-SQL script used for parallel cleaning of NGS data in performed experiments.

During our tests, we used the raw NGS data obtained from the NGS sequencing of the *Drosophila melanogaster* with the paired-end method. We tested our library on four NGS data sets (each providing two FASTQ files containing the corresponding sequence reads from the 3' to 5' end of the sequenced DNA fragment). The data were collected from the Sequence Read Archive database (Leinonen et al., 2010). The following NGS data sets were used in our experiments:

- SRR988072 (two files, 4.95 GB each),
- SRR988073 (two files, 3.61 GB each),
- SRR988074 (two files, 5.2 GB each),
- and SRR988075 (two files, 11.7 GB each).

The total amount of data was about 50.1 GB for uncompressed data. For the compressed data (gzip-based compression), the total amount of data was ~14.8 GB (SRR988072—1.48 and 1.30 GB, SRR988073—1.08 and 951 MB, SRR988074—1.62 and 1.45 GB, and SRR988075—3.63 and 3.33 GB). These files contained the NGS data characterized by low quality and contamination with Illumina adapters. For this reason, they were selected for our tests related to NGS sequence cleaning.

### 3.1. Processing Multiple Genomes

One of the advantages of the Data Lake ecosystem is the possibility of processing the NGS data of many genomes simultaneously. In this section, we present the results of performance experiments carried out for parallel processing of all data sets (SRR988072, SRR988073, SRR988074, and SRR988075) for various storage scenarios, file formats, and compression used. Experiments were performed with 8 AUs. For this experiment, the NGS sequence reads were stored in their native FASTQ files and the row-oriented files. Additionally, we also divided the NGS data into 250 MB FASTQ files to increase the level of parallelism (manually apply the medium-grained parallelism) and verify whether it affects the performance of the EPS process. The 250 MB size of the files fits exactly one block of data, called an extent, assigned to a single unit of parallelism—a vertex in the execution graph.

**Figure 4:**
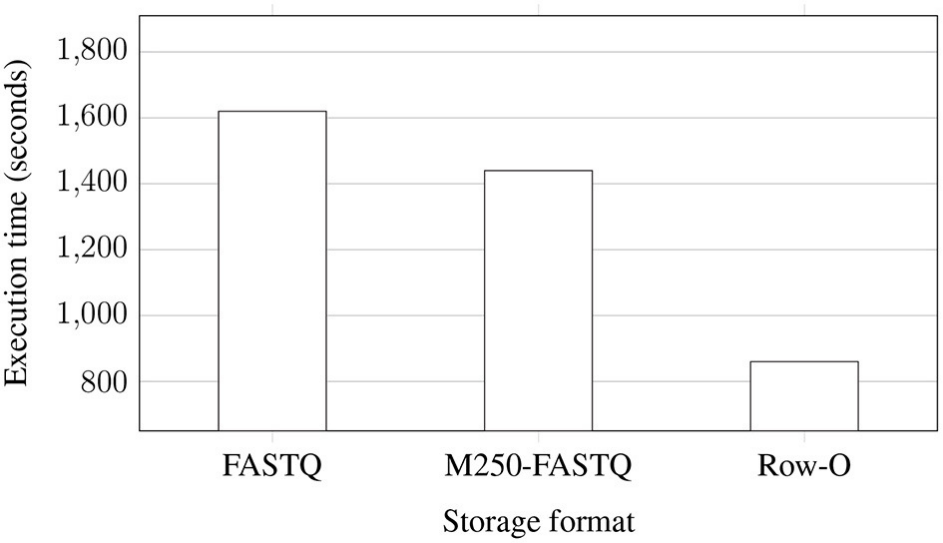
Processing times of NGS data extracted from uncompressed files and saved to eight uncompressed FASTQ files (two files for each of the processed genomes) with 8 AUs for various storage formats: regular FASTQ, multiple 250 MB FASTQ (M250-FASTQ), and row-oriented (Row-O).

Figure 4 shows the execution time of the whole EPS process performed for improving the quality of NGS data stored in the three formats. The data were extracted from uncompressed data files and stored again in uncompressed files after improving the quality. As can be observed, processing the whole FASTQ files takes the longest, while row-oriented files are processed almost two times faster. The distribution of data to multiple FASTQ files brings some improvement, but it is not huge.

**Figure 5:**
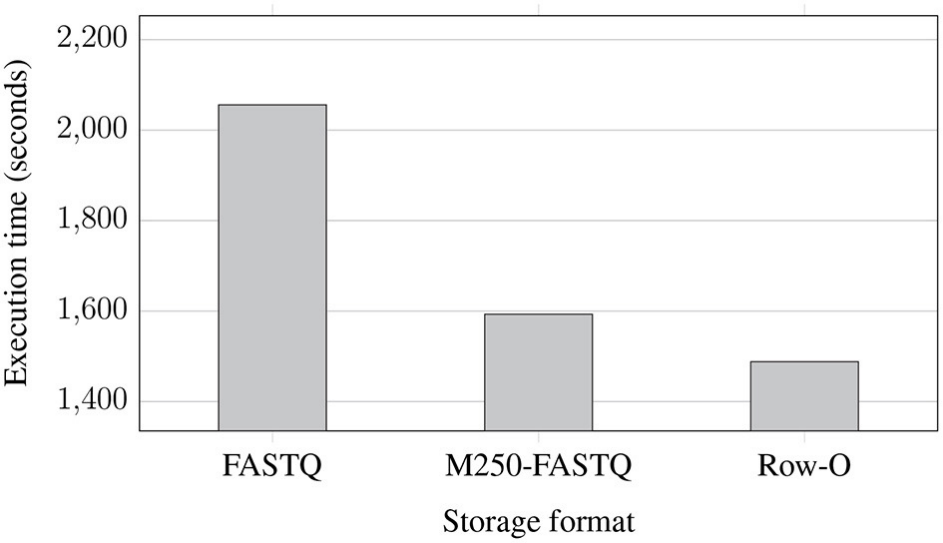
Processing times of NGS data extracted from compressed files and saved to eight compressed FASTQ files (two files for each of the processed genomes) with 8 AUs for various storage formats: regular FASTQ, multiple 250 MB FASTQ (M250-FASTQ), and row-oriented (Row-O).

Figure 5 shows the execution time of the whole EPS process performed for improving the quality of NGS data stored in the same three formats. However, in contrast to the previous experiment, the data were extracted from compressed data files and stored in compressed files after improving the quality. In terms of storage format, conclusions are similar to those from the previous experiment. However, we can observe that for the compressed data, the use of the row-oriented format does not bring such a huge improvement in the execution time. Comparing the results of both experiments, we can also notice that processing the compressed data takes more time, which is caused by additional decompression and compression steps while extracting and storing the data in the EPS process.

**Figure 6:**
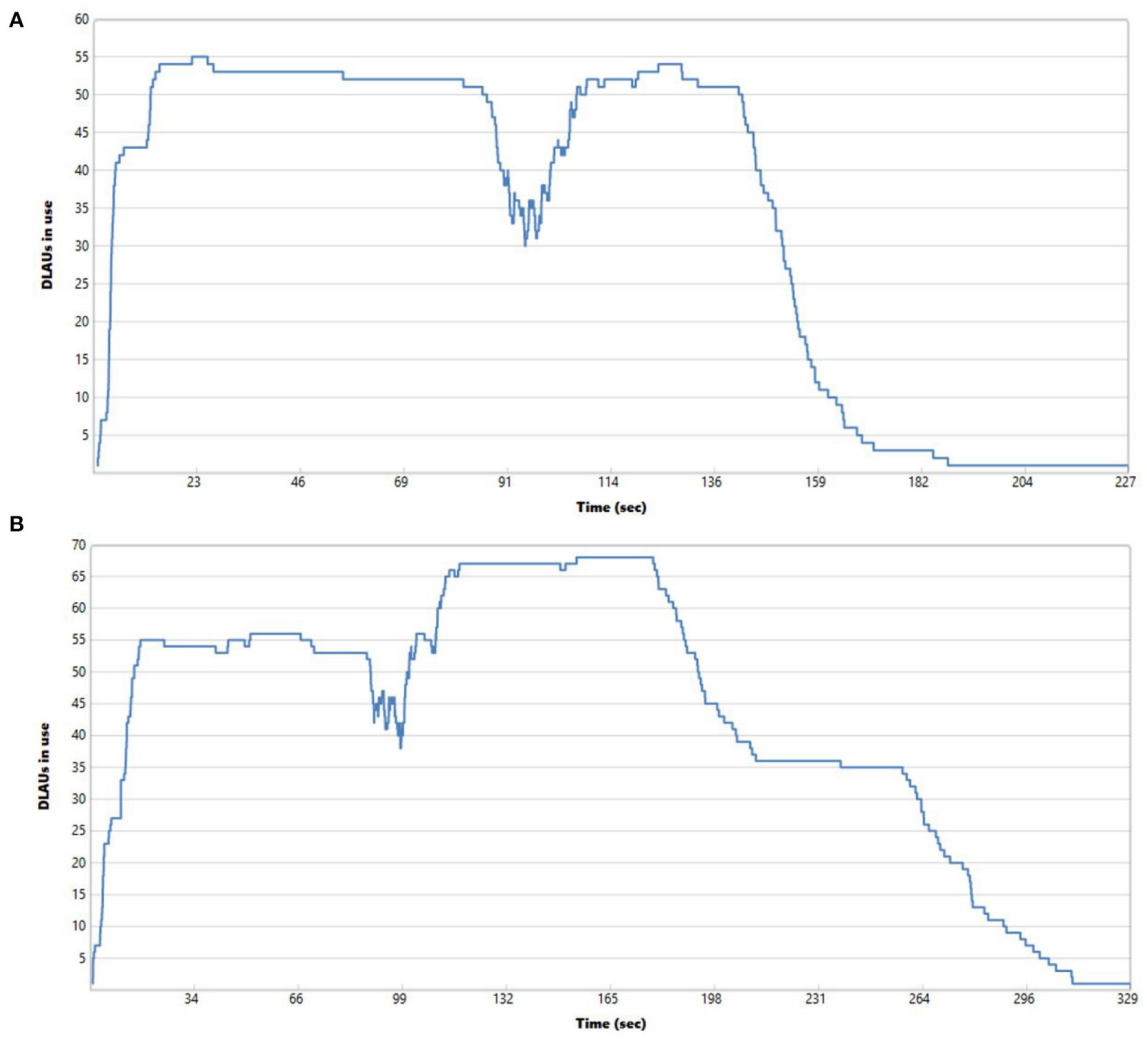
Utilization of Allocation Units (computational units) over time while processing data from four uncompressed **(A)** and compressed **(B)** row-oriented files with forward and reverse reads and saving to eight uncompressed **(A)** and compressed **(B)** FASTQ files (two files for each of the processed genomes) with 55 AUs **(A)** and 68 AUs **(B)**.

It is worth noting that in both cases of processing compressed and uncompressed files, the row-oriented format turned out to be highly scalable. When scaling out to many AUs for the same collection of data, we could decrease the execution time to 227 s when processing uncompressed row-oriented files and to 329 s when processing compressed row-oriented files for all data sets and storing the cleaned NGS data to eight uncompressed and compressed FASTQ files in both scenarios. Figure 6 shows the utilization of Allocation Units over time while processing the data. It can be observed that AUs are not evenly utilized during the whole execution time. Especially in the final phases of the job execution, their utilization is low due to storing in FASTQ files, for which we cannot rely on the medium-grained parallelism.

### 3.2. Performance Gain Over Local Processing

In the next series of experiments, we compared the execution time of the whole EPS process performed in the Data Lake environment and on local workstations. The data processing time on the local computers was checked using two machines with different configurations. The first workstation had an Intel Core 2 Duo 3.6 GHz CPU, 3 GB DDR memory, and 320/7200/16 hard disk drive. The second workstation had much better compute capabilities. It was equipped with an Intel Core i7-4790K 4 GHz processor, 16 GB DDR3 memory, and the same type of hard disk drive. For improving the quality of data, we used the original Trimmomatic program. NGS data were processed to achieve the best possible quality scores. The following commands were used during the data processing phase for particular NGS data sets (SRR988072, SRR988073, SRR988074, and SRR988075), analogous to those used to perform data cleaning in the Data Lake ecosystem:

>ILLUMINACLIP:TruSeq3-PE.fa:2:30:10 LEADING:20 TRAILING:20

 SLIDINGWINDOW:4:20 MINLEN:30

>ILLUMINACLIP:TruSeq3-PE.fa:2:30:10 LEADING:20 TRAILING:20

SLIDINGWINDOW:4:20 MINLEN:30

>ILLUMINACLIP:TruSeq3-PE.fa:2:30:10 TAILCROP:10 LEADING:20

TRAILING:20 SLIDINGWINDOW :4:20 MINLEN:30

>ILLUMINACLIP:TruSeq3-PE.fa:2:30:10 TAILCROP:10 LEADING:20

TRAILING:20 SLIDINGWINDOW :4:20 MINLEN:30

**Figure 7:**
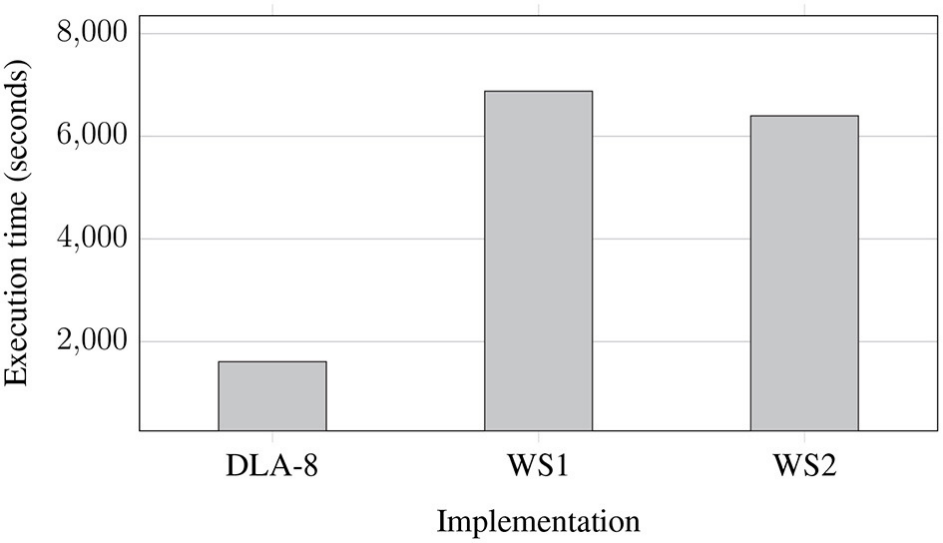
Processing times of NGS data extracted from regular uncompressed FASTQ files and saved to eight uncompressed FASTQ files (two files for each of the processed genomes) for various implementations: on Data Lake with 8 AUs (DLA-8), workstation 1 (WS1), and workstation 2 (WS2).

Figure 7 shows processing times for NGS data extracted from regular uncompressed FASTQ files and saved to eight uncompressed FASTQ files (two files for each of the processed genomes) for various implementations: on Data Lake with 8 AUs (DLA-8), workstation 1 (WS1), and workstation 2 (WS2). This experiment shows a pessimistic case since whole FASTQ files are processed at the coarse-grained level of parallelism. As we can observe, the processing time was reduced more than three times in the Data Lake environment. It is not linearly proportional to the number of AUs in use (8 AUs), but differences in sizes of processed FASTQ files and granularity of parallelism do not allow for better performance gain (some AUs finish their processing earlier and stay idle for some time, as it was presented in Figure 3).

### 3.3. Quality Control

**Figure 8:**
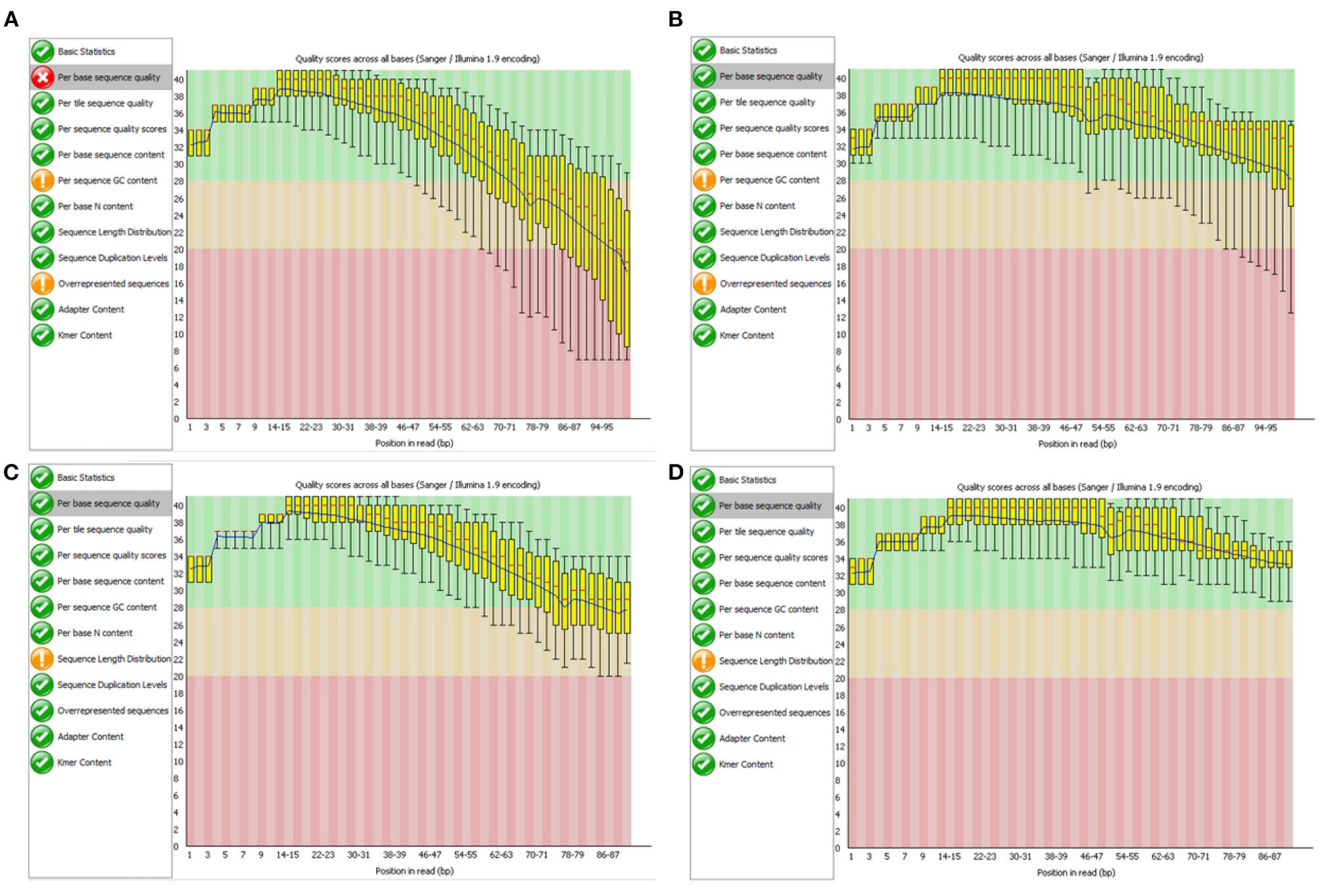
Evaluation of nucleotide sequence quality in the FASTQ files SRR999074_1 **(A,C)** and SRR999074_2 **(B,D)** before **(A,B)** and after **(C,D)** improving the quality with the developed software.

Our library was created to allow scalable processing and improving the quality of NGS raw data stored in the Big NGS Data Lake. At the same time, the library implements the set of functionalities of the Trimmomatic application, an open-source desktop program intended for this purpose. As a part of our experiments, we also validated the effectiveness of our library in terms of the quality of results. Tests were performed for all genome sequences in our NGS Data Lake. In this section, we show the validation results on the example of NGS data marked with the SRR988074 identifier. To validate the effects of the cleaning process performed on FASTQ files with our library for data lake, we used the FastQC tool (Wingett and Andrews, 2018). Figure 8 shows the comparison of results generated by the FastQC program presenting the assessment of the quality of nucleotides in DNA sequence reads from two NGS files storing data obtained with the paired-end sequencing technique. We can observe that quality scores of the sequence reads before the cleaning process drop below 20 (for the file with forward reads SRR988074_1, Figure 8A). After the cleaning process, the quality scores stay above 25 (Figure 8C), and even 30 for the file containing reverse reads (SRR988074_2, Figure 8D). In the case of both files, definite improvement is visible.

Since we implemented the same set of functionalities as in the Trimmomatic, in terms of the quality improvement, the results are the same as for the original desktop application.

## 4. Discussion and Future Directions

Improving the quality of NGS data is one of the first steps preceding the secondary analysis of DNA genome sequences and further NGS-based analyses. Our work confirms that the steps of the pre-processing can be performed on a large scale by (1) collecting the massive volumes of NGS data in the NGS data lake, (2) processing them in parallel within the EPS process, and (3) scaling the computations in the Cloud. Our library becomes then a handy element of the early stages of the secondary analysis of NGS data.

Although, as we could see, processing some storage formats (like the whole compressed FASTQ files) do not provide linearly proportional performance gain and do not allow utilizing both types of parallelism, we also found a way to parallelize computations for other storage formats efficiently (e.g., whole native, uncompressed FASTQ files) and take advantages of the platform capabilities and the techniques we propose. Our experiments showed that we could benefit from the coarse-grained parallel processing when we process multiple genomes. Medium-grained parallelism is advantageous mainly for row-oriented files. However, our solution has limitations for handling the whole FASTQ files, for which the medium-grained parallelism cannot be applied in all phases of the EPS process.

Our experiments also showed that to take full advantage of such data lake platforms, it is advisable to work with data split into many smaller files or work with non-standard formats for storing the NGS data, like the row-oriented format presented in the paper. However, this would require either additional data pre-processing (to split the data) or changing the formats in which data are provided for analysis. The row-oriented format is the best-fitting one for all Big Data platforms and would give the best performance.

Our library complements other solutions presented in section 1 by providing a set of functionalities that are dedicated to cleaning NGS data on a large scale in a highly scalable environment, which was not available so far. In such a way, it extends the data cleaning capabilities of the Trimmomatic package toward large data sets. Like SeqPig and GMQL, the functionality of our library is exposed through a declarative language, but for a different purpose. Also, in our project, we used the U-SQL language that combines capabilities of the SQL language used for querying relational databases with the C# programming language, which is highly extendable. However, the limitation of the adopted Data Lake platform is that it is tightly linked with the Azure cloud. Therefore, unlike the SeqPig or SeQual, it is not portable to other cloud platforms. On the other hand, our Data Lake library allows improving the quality of NGS data obtained with the use of single-read and paired-end sequencing techniques and stored in various formats, which are also significant unique features of our solution.

## 5. Concluding Remarks

Secondary and tertiary analyses performed by scientists working in genomics and computational biology require high-quality data and an infrastructure that provides appropriate space to store large amounts of data generated as a result of next-generation sequencing techniques at various stages of the analysis. Data obtained from single genome sequencing can reach up to several hundred gigabytes and may be of various quality. The infrastructure used to analyze the NGS data should also provide computing power to allow rapid and scalable processing of gathered data. The hybridization of tools that enable handling computations over big biomedical data with extensive scaling capabilities of the Cloud proved to be a reasonable solution. This work shows the successful implementation of the early stage pre-processing techniques used in NGS data analysis pipelines in a distributed environment of the Data Lake ecosystem hosted in the Microsoft Azure cloud computing platform. Data Lake Store allows hosting data in any format and without restrictions on the amount of data stored. These characteristics make Data Lake a perfect place for storing large amounts of data for further analysis. In such a way, our solution addresses the *volume* and *variety* challenges of processing Big NGS Data. The Data Lake Analytics allows then for parallel processing of many genomes simultaneously in a distributed environment, addressing the *velocity* challenge. This would be difficult to achieve on, for example, desktop computers due to the limited capabilities of the processors or hard disk drives. Additionally, the use of the Data Lake Analytics and serverless computing paradigm reduces the maintenance overhead and removes the need to maintain and scale underlying computing clusters manually. Finally, procedures and functions for improving NGS data quality embedded in declarative U-SQL queries simplify the cleaning process that ultimately leads to the increase in the *value* of obtained results.

## Data Availability Statement

Publicly available datasets were analyzed in this study. This data can be found at: Sequence Read Archive (https://trace.ncbi.nlm.nih.gov/Traces/sra/?run=SRR988072).

## Author Contributions

DM proposed the idea and KS extended it. DM conceived and designed the experiments and prepared the experimental environment. KS and DM performed the experiments, verified results, and designed and implemented the tools. DM and BM-M analyzed the data. PG reviewed the related literature. BM-M, KS, PG, and DM wrote the paper and made revisions to address comments of the referees. All authors contributed to the article and approved the submitted version.

## Conflict of Interest

The authors declare that the research was conducted in the absence of any commercial or financial relationships that could be construed as a potential conflict of interest.
